# Evaluation of the Effect of Post-Processing Methods on the Surface Parameters of Parts Produced by FFF/FDM Technology

**DOI:** 10.3390/ma18204672

**Published:** 2025-10-11

**Authors:** Marek Kočiško, Lukáš Štafura, Karol Goryl, Zuzana Mitaľová

**Affiliations:** Faculty of Manufacturing Technologies, Technical University of Kosice, Bayerova 1, 080 01 Presov, Slovakia; lukas.stafura@student.tuke.sk (L.Š.); karol.goryl@tuke.sk (K.G.); zuzana.mitalova@tuke.sk (Z.M.)

**Keywords:** postprocessing, roughness, layer, composite, sandblasting

## Abstract

This article focuses on evaluating selected roughness parameters on samples created by material extrusion, specifically FFF (Fused Filament Fabrication). The experiment was divided into two separate phases. The first phase of the experiment involved creating a four-level model A from PLA (poly (lactic acid)) material without any additives. The variable parameter was the height of the printed layer, where each level was printed at a different print height. Subsequently, the sandblasting method was applied to the samples using a selected abrasive. The roughness parameters were evaluated using a Mitutoyo Surftest SJ-400 roughness tester. Based on the results of the roughness parameters of model A, model B was prepared, using a constant print height. Each level of model B was made with different metallic additives based on PLA material. The findings demonstrate the effectiveness of mechanical post-processing in achieving the desired surface characteristics of additively manufactured components. The experiment confirms the suitability of sanding and grinding to improve surface quality at different layer heights and for PLA-based materials with metal additives. In addition, grinding and sanding of PLA-based composites filled with metal particles can create a realistic metallic appearance comparable to conventionally manufactured metals.

## 1. Introduction

Additive manufacturing is a process of creating a physical object based on digital modeling data, which involves the successive deposition of individual layers of material. The object is digitally sliced into layers, which are then gradually deposited on top of one another. In the early stages of this technology’s development, additive manufacturing was carried out using large industrial equipment, whereas three-dimensional (3D) printing was applied on a smaller scale. Today, these terms are often used interchangeably [1,2].

Additive technologies today represent a significant advancement in the design and production of components with structurally and geometrically complex shapes. In general, these technologies can be classified into three groups based on the material deposition method, the physical state of the applied material, and the medium used during production. The most commonly used technologies include ME (Material Extrusion), SLA (Stereolithography), and PBF (Powder Bed Fusion) [1,2].

Material Extrusion (ME), also commercially known as Fused Deposition Modeling or Fused Filament Fabrication, is one of the most widely used additive technologies. The quality of the manufactured parts is fundamentally influenced by the temperature profile of the cooling process, which can lead to deformations, waviness or the formation of porosity. The temperature of the extruder and the already deposited layer is also a critical factor, as the quality of the bond between the individual layers and thus the mechanical properties of the final part depend on it. Anisotropy of mechanical properties is a significant problem, since the orientation of the deposited layers creates differences between the strength in the XY plane and in the Z axis. Modeling in the field of ME therefore focuses on thermal phenomena, dimensional accuracy, production speed and surface roughness. Studies show that parameters such as layer thickness, build orientation, raster angle, air gap or scanning speed have a direct impact on tensile, bending and impact strength, but also on residual stresses and surface quality [3,4].

Material extrusion operates on the principle of gradually depositing layers of thermoplastic material, which can lead to imperfections in the form of surface irregularities and unwanted textures on the printed objects. The formation of surface roughness during the printing process compromises the visual appearance of samples and negatively affects the mechanical properties of the printed components. Based on this fact, there is an ongoing effort to eliminate undesired surface structures resulting from the application of the Fused Deposition Modeling method; post-processing represents a critical step to ensure products meet necessary structural, material, and aesthetic requirements [4,5].

The main objective of this article is to apply and verify surface treatment methods aimed at removing or reducing undesirable surface structures, such as layer lines, spatter, or droplets, which are characteristic of material extrusion technology in order to achieve the desired surface properties. The innovation of the article lies in demonstrating that mechanical post-processing methods, specifically sandblasting and grinding, can significantly improve the surface properties of parts printed using Material Extrusion (FFF) even at increased layer heights, thereby increasing printing productivity without compromising surface quality. In addition, the work demonstrates that PLA composites filled with metal particles acquire a realistic metallic appearance during post-processing, opening up new possibilities for use in design and prototyping applications where aesthetics are critical but the cost and complexity of traditional metal manufacturing would be prohibitive.

## 2. State of the Art

Most models produced using material extrusion technology exhibit significant surface irregularities, which require additional surface finishing [2]. In conventional manufacturing, surface treatment technologies are categorized according to the technique used (a brief overview is provided below). However, not all available technologies are applicable for surface treatment of parts produced by material extrusion technology. This experiment mostly focuses on the mechanical surface treatment method—sandblasting. Metal-filled PLA composites offer unique opportunities for metallic finishes [6,7].

Chemical surface treatment—one of the most commonly used chemical methods is vapor smoothing. The entire process takes place in a closed chamber containing the model, a container with acetone, and a fan. The connected fan circulates the air, causing the acetone to evaporate. This process gently liquefies the part’s surface, filling in imperfections and creating a uniform glossy finish. It must be noted, however, that this method is not effective for all commonly used filament types. Acetone is not an effective agent for PLA material, while on the other hand, it is effective for ABS. A drawback of this treatment is the requirement for a sufficiently ventilated area or filtration system (extraction) [2,8,9].

Rolling and shot peening are effective methods of mechanical surface treatment, in which rollers or balls impact the model’s surface, leading to its hardening, increased surface hardness, and the creation of a relatively smooth finish [8,10,11].

Stavropoulos et al. focused on the challenges of industrial application of additive manufacturing and proposed a hybrid solution framework that combines additive and subtractive processes. The authors analyzed the limitations of AM, in particular low productivity, the need for additional processing, limited working volume, and unpredictable part errors. As part of the study, they presented the concept of a modular hybrid manufacturing system based on robotic arms, which allows the combination of processes such as metal powder melting (DED, WAAM) with machining, laser processing, or grinding. The solution also includes process monitoring using sensors, adaptive control, and a digital twin, which should lead to higher accuracy, flexibility, and productivity. The results show that hybrid systems can significantly reduce production times, lower costs, and improve surface quality compared to standalone AM technology. For future research, the authors emphasize the need to verify the reliability of cyber-physical systems in industrial applications and to develop control strategies for integrated AM-SM lines [12].

Alex Quok An Teo et al. focused their research on the post-processing of samples produced using additive manufacturing for biomedical applications. Specifically, they designed a lattice structure with dimensions of 4.25 × 4.25 × 3 mm^3^ made from austenitic stainless steel AISI 316L, using the Selective Laser Melting (SLM) technology. They proposed three finishing operations to improve surface quality: sandblasting, abrasive polishing, and electrolytic polishing. To evaluate the surface of the samples, digital microscopy (DM) and scanning electron microscopy (SEM) were employed, where surface roughness parameters were measured and the overall surface topography was assessed. Imperfections caused by the material extrusion process were effectively removed by sandblasting. The average values of Ra and Rz after sandblasting were 5 µm and 37 µm. After mechanical polishing, the roughness values were reduced by an additional 2 µm compared to sandblasting. However, the polishing process introduced undesirable effects on the surface integrity—such as the formation of surface depressions. For comparison, electrolytic polishing was also applied. The achieved values of Ra and Rz ranged between 37–40 µm. In conclusion, the authors recommended continuing research, particularly in relation to applying other potential finishing operations [13].

Souflas et al. conducted a comparative study of dry and cryogenic milling of components made of Inconel 718 nickel alloy, manufactured using Directed Energy Deposition (DED) technology. In the experiment, they tested the effect of liquid nitrogen (LN_2_) as a cooling medium during milling and evaluated cutting force, tool wear, surface roughness, and residual stress. The results showed that cryogenic cooling significantly reduced cutting force (by up to 47% in the feed axis) and tool wear (by 40–50%), while promoting even wear of the cutting edges. Surface roughness remained at the submicrometer level for both cooling methods, although it was slightly higher for cryogenic machining. Residual stress analysis confirmed that cryogenic cooling causes higher surface stresses, which is attributed to the surface hardening effect. The authors concluded that cryogenic milling is a promising alternative for the hybrid production of parts from difficult-to-machine alloys, as it reduces tool forces and wear while allowing machining at higher cutting speeds. For future research, they recommend a more detailed investigation of the interaction of cryogenic cooling with cutting process parameters and its effect on surface integrity in additively manufactured metal parts [14].

Kariž M. et al. focused their experiment on the strength of joints between extruded samples and beech wood, where surface modification techniques were used to influence bond strength. The materials used for prepared parts were PLA, PLA with wood-based filler in particle form, and ABS. PVAc adhesive was used for bonding with beech wood samples, and the printed part dimensions were 50 × 20 × 10 mm. The authors compared the adhesion of samples with untreated surfaces, sanded surfaces, plasma-treated surfaces, and a combination of sanding and plasma treatment. Laser microscopy and scanning electron microscopy were used for evaluation. The bonded joints were subjected to tensile and shear stress tests. Initial results showed that sanding significantly improved adhesion, whereas plasma treatment alone led to melting of the material, reducing bonding quality. The combination of both technologies yielded the highest adhesion between bonded surfaces. For PLA material without filler, the achieved bond strength was 3.72 MPa; for PLA filled with wood particles, the bond strength reached 3.96 MPa. In testing, the ABS material achieved the highest bond strength—4.83 MPa—when surface treatments were combined. In their conclusion, the authors emphasize the need for further research, recommending the fabrication of larger test samples [15].

Žigon J. et al. focused their study on surface treatment of prepared samples through the application of various coatings. The fabrication method used was material extrusion, and the samples (150 × 70 × 4 mm) were made from three materials: PLA, wood-filled PLA, and ABS. Before applying the prepared coatings, the sample surfaces were gently sanded using P120 grit sandpaper. The applied coatings included: a dissolved alkyd coating, a water-based acrylic coating, and a coating made from ABS material dissolved in acetone. Adhesion was evaluated in accordance with ISO 4246 [16], which defines the testing conditions for coating adhesion. Wettability was observed on the coated samples, and the adhesion of the applied layers was subsequently tested. The highest adhesion was achieved with ABS material coated with ABS dissolved in acetone, reaching values between 2.5 and 2.9 MPa. The wood-filled PLA samples also showed relatively high adhesion, attributed to the presence of microscopic wood particles and the resulting increased surface porosity. This porosity allowed the coating to penetrate deeper into the structure of the sample [17].

Mathew A. provided a comprehensive overview of surface treatment methods for samples produced via additive manufacturing, specifically using the material extrusion technique and materials PLA and ABS. The study described basic surface treatment approaches—including mechanical, chemical, and thermal methods—as well as their combinations. The focus was placed primarily on chemical surface treatment. The surface of the samples was exposed to solvent vapors, specifically acetone and tetrahydrofuran (THF), to observe the effects on surface quality, mechanical properties, and porosity. PLA samples showed the lowest surface roughness values after exposure to THF vapor for 5 min at 65 °C. However, this treatment resulted in an approximate 10% reduction in mechanical properties. For ABS, the lowest surface roughness was observed after exposure to acetone vapor for 45 min. Beyond this exposure time, mechanical properties deteriorated significantly due to increased brittleness of the surface layer. Chemical treatment with solvent vapors proved to be effective for these materials. By optimizing temperature and exposure time, it is possible to significantly improve surface quality while maintaining the required mechanical performance [18].

## 3. Materials and Methods

### 3.1. Used Materials and Sample Preparation

This section of the article describes the materials used for samples, the design of extruded models, the production process, and the setup of printing parameters. In the first phase of the experiment, samples were printed from pure PLA (without additives), with the goal of examining the influence of printed layer height (ranging from 0.22 to 0.52 mm) on surface roughness parameters Ra and Rz. This was followed by the selection of a suitable post-processing method and the implementation of the second experimental phase. In the second phase of the experiment, samples were made using PLA blended with various metal fillers—iron, steel, and bronze—materials commonly applied in design and for creating visual prototypes. Based on the results of the first experimental phase with model A and the printing speed, a constant layer height of 0.52 mm was chosen for model B. The sample geometry remained unchanged. Surface roughness parameters Ra (Arithmetic Mean Deviation) and Rz (Maximum Height of Roughness Profile) were again evaluated before and after post-processing.

The material used in the first phase was PLA supplied by Protopasta, and the resulting extruded sample was designated as Model A. PLA was chosen for its ability to print at relatively low temperatures (180–220 °C) without the need for a heated bed, and it is the most commonly available filament. The materials used in the second phase included PLA, PLA with iron additive, PLA with steel additive, and PLA with bronze additive from the manufacturer Protopasta. The extruded sample was designated as Model B. The specific properties of the applied materials are provided in Table 1.

**Figure 1:**
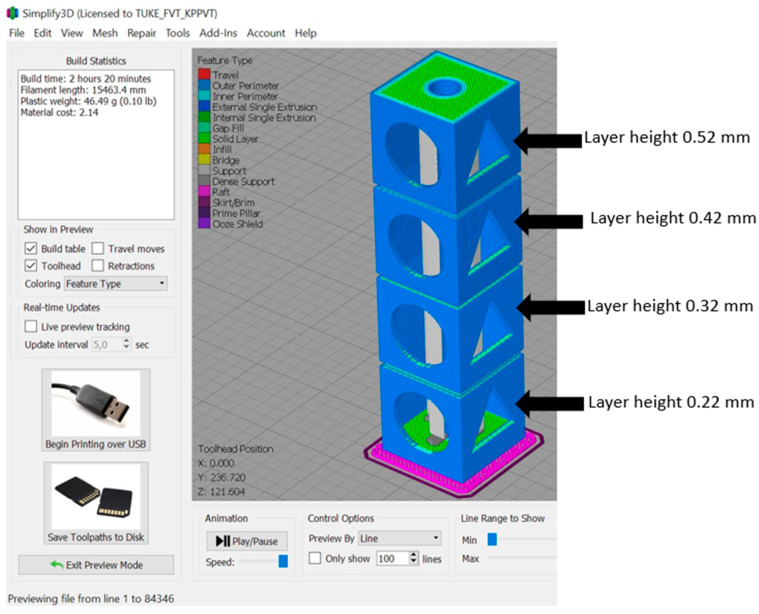
Model A with layers visualized in the slicing software Simplify.

**Figure 2:**
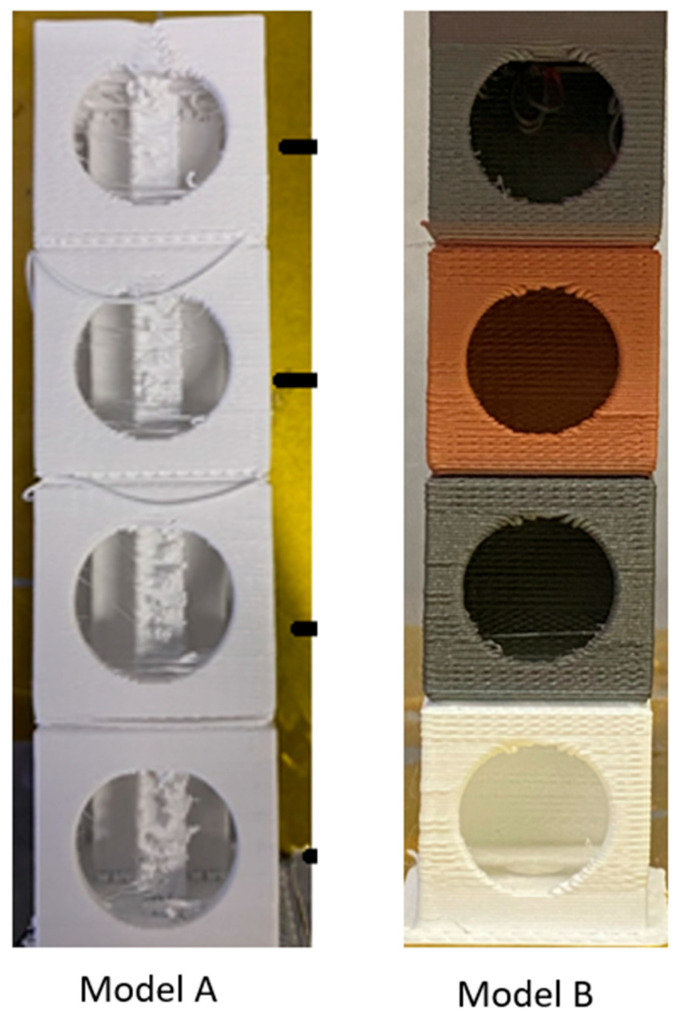
Model A versus Model B with retained support structures and imperfections.

Geometry of models: base 30 × 30 mm; total height 120 mm (four-level model). Models were created using CAD software Creo Parametric 10.0. Two types of holes were designed on the models, positioned as shown in Figure 1, in the shapes of a circle and a triangle. The holes were intentionally created to monitor changes and capture surface irregularities/deformations after the process. The prepared STL file was sliced using Simplify3D 4.1.0 software (a tool for setting printing parameters), and printing was performed on a Creality Ender 3 V2 NEO. The input printing parameters were selected based on the material sheets provided by the filament manufacturer along with an overview of previously performed experiments and are listed in Table 2. For model B, a printing layer height of 0.52 mm was applied to save time. Specifically, printing with a layer height of 0.22 mm took 5 h and 22 min, while printing with a layer height of 0.52 mm took 2 h and 26 min and can be seen in Figure 2.

### 3.2. Used Method for Surface Finishing

**Figure 3:**
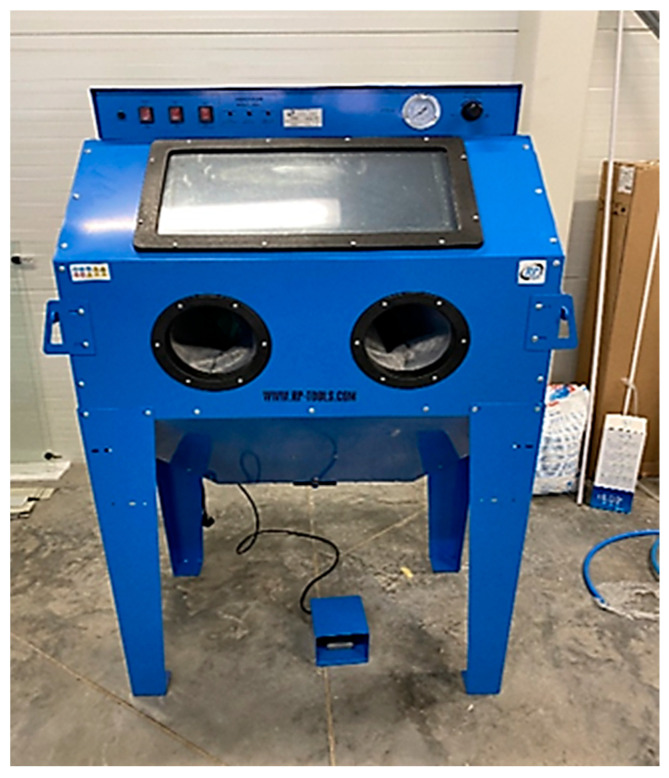
Sandblasting cabin RP-SG350L with a volume of 350 l.

During the sandblasting process, an RP-SG350L sandblasting cabin (RP-TOOLS company) with a volume of 350 L (Figure 3) was used in 30-min cycles at a pressure of 6 bar. Three types of abrasives were applied in the sandblasting process, specified below:

- Australian garnet—a sharp-edged blasting medium containing ferrite, suitable for sandblasting steel, stainless steel, iron metals, wood, glass, marble, and similar materials. It is characterized by high hardness (7.5–8.0 Mohs) and is reusable 5 to 10 times without loss of cutting ability.
- Silica sand—characterized by a high quartz content and medium grain size, suitable for industrial blasting. The grains of this medium are isotermic, well-rounded, highly durable, and chemically stable.
- Plastic grit—a hard material resistant to scratching, with good chemical/thermal resistance and electrical properties.

**Figure 4:**
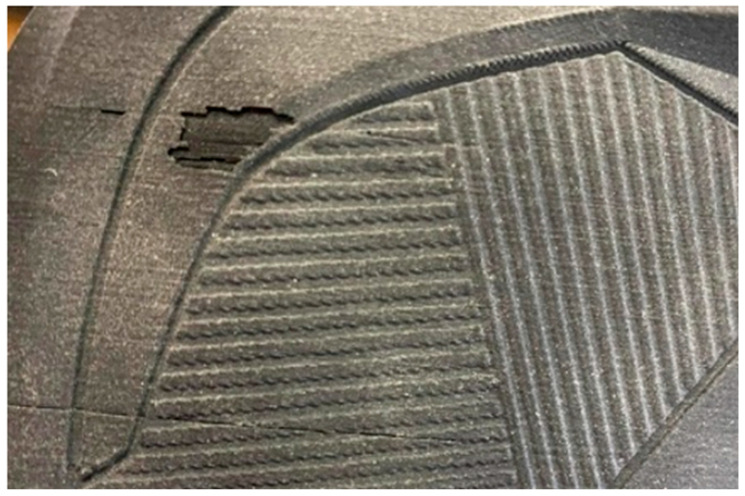
Damaged surface of the model after sandblasting with plastic grit application.

During the application of plastic grit, significant problems occurred during sandblasting—layer delamination of the model and clogging of the blasting gun. Similar issues were observed with the use of Australian garnet. For this reason, the experiment was subsequently limited to one type of blasting medium—silica sand (Figure 4).

**Figure 5:**
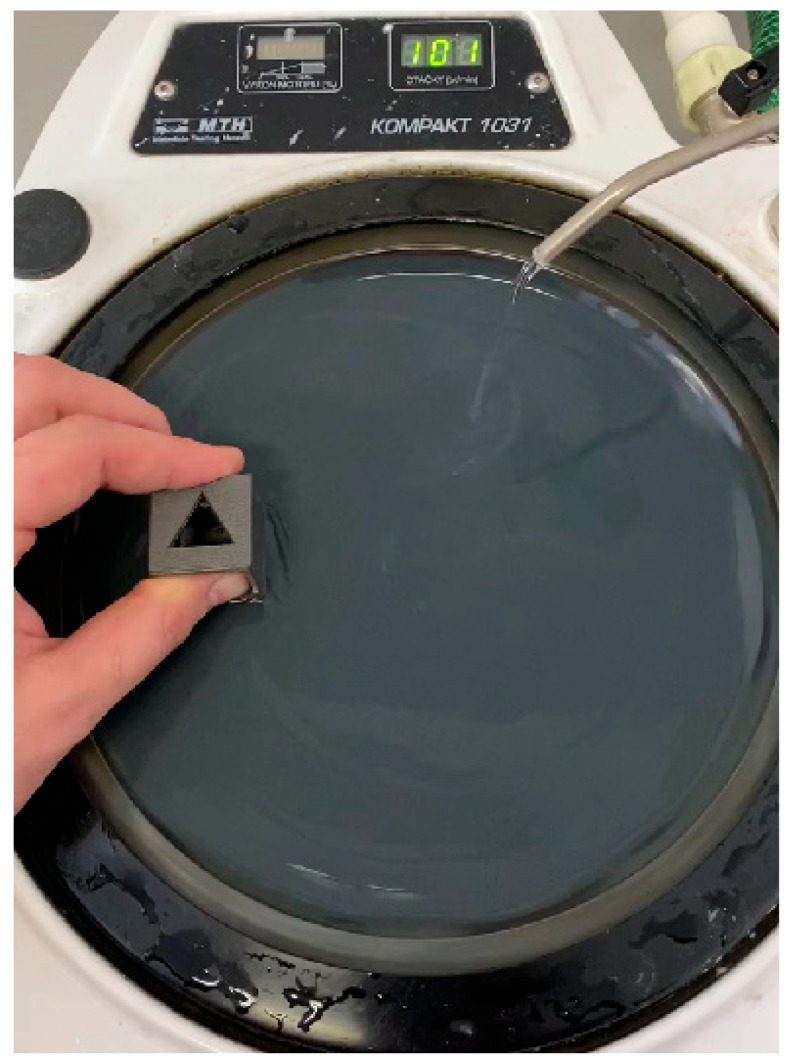
Metallographic water-based polishing.

Also, water-based sandpaper was used as a secondary operation for surface treatment to achieve lower surface roughness and better results for presentation purposes. Subsequently, secondary processing of model B was performed by sanding. The sanding process was carried out stepwise using sandpapers with grit sizes of 600, 2000, and 4000 (abrasive grain type: SiC) on a manual double-disc sander for metallographic polishing, model QPOL 250 M2. Cooling with water was applied during the process due to the thermal sensitivity of the PLA material. Without cooling during sanding, PLA material experiences excessive overheating, localized melting, and deformations. The surface roughness parameter values after sanding are shown in Figure 5.

The results of these measurements and comparisons will help determine the optimal conditions for using PLA with various fillers, enabling better adaptation of the material for specific applications such as design and the creation of visual objects.

## 4. Results and Discussion

For quantitative evaluation of surface roughness, the standard STN EN ISO 4287 [19] was applied. Surface roughness is a geometric property, and there are no direct methods for its assessment. Instead, appropriate characteristics and parameters, considered as criteria of surface roughness (measured in micrometers, µm), are measured. The evaluation criteria used were: Rz and—both assessed relative to the baseline over the evaluation length lr.

**Figure 6:**
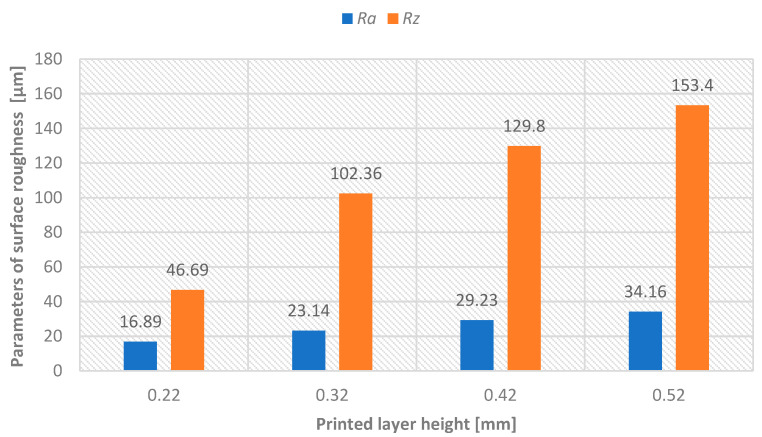
Graphical representation—printed layer height versus achieved surface roughness parameters Ra and Rz before sandblasting surface treatment.

**Figure 7:**
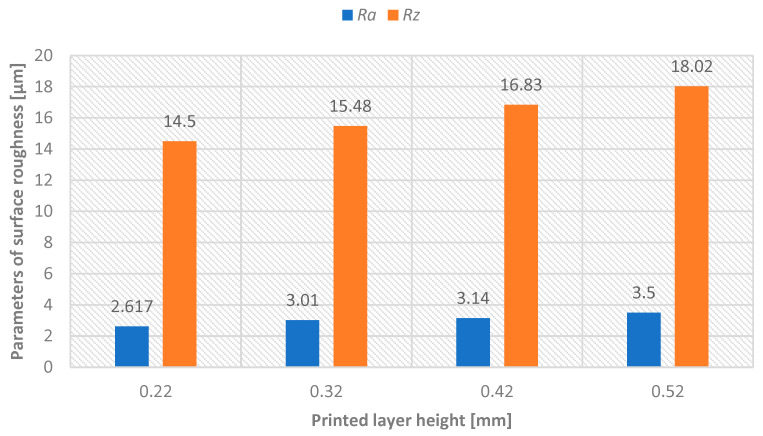
Graphical representation—printed layer height versus achieved surface roughness parameters Ra and Rz after sandblasting surface treatment.

For measuring the surface roughness parameters, the MITUTOYO Surftest SJ-400 device was used, equipped with automatic compensation of radius and tilt, designed for contact (tactile) measurement of roughness, waviness, and primary profile. The evaluation length was lr = 4 mm, with a profile filter λc = 0.8 mm (λs = 2.5 μm). Values of Ra and Rz parameters were measured with a repeatability of 32 times. To exclude values affected by gross errors, the Grubbs test (at a 95% confidence level) was applied. The percentage comparison is summarized in the Table 3. Subsequently, arithmetic means of Ra and Rz parameters were calculated and used in graphical representations. Before surface treatment of model A, surface roughness parameters were measured for each printed layer height—see Figure 6. After surface treatment of model A, surface roughness parameters were measured for each printed layer height—see Figure 7.

**Figure 8:**
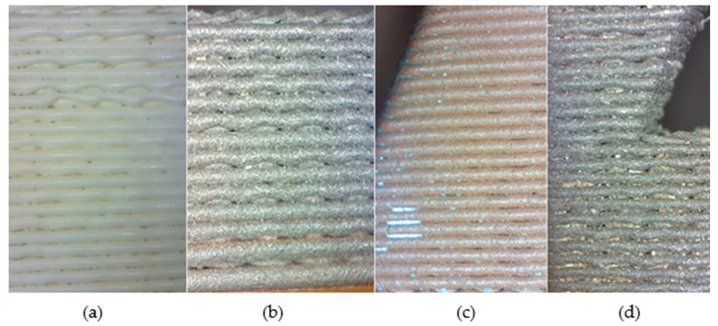
Details of Model B: (**a**) PLA, (**b**) PLA Iron, (**c**) PLA Bronze, (**d**) PLA Steel at 10× magnification.

Model B—printed with an emphasis on the materials used, with a layer height of 0.52 mm. Defects are visible in the model even at 10× magnification—these include gaps in the walls and material leakage—see Figure 8. The part made of PLA material with copper additive shows the first signs of corrosion, which is a desirable visual feature of the filament.

The experimental results demonstrate that post-processed metal-filled PLA composites can achieve visual characteristics indistinguishable from conventionally manufactured metals. This finding has significant implications for design applications, architectural prototyping, and decorative components where authentic metallic appearance is required without the cost and complexity of metal processing. The controlled oxidation observed in PLA Iron and Bronze composites represents a desirable aesthetic feature for design applications, creating authentic weathered metal appearances that would typically require years of natural aging or expensive chemical treatments on real metals.

For Model A, increasing layer height from 0.22 mm to 0.52 mm resulted in a significant deterioration of surface roughness, with Ra values increasing from 16.89 μm to 34.61 μm (105% increase) and Rz values rising from 76.69 μm to 153.42 μm. This quantitative data supports the fundamental trade-off between production speed and surface quality in material extrusion technology.

Sandblasting with silica sand demonstrated exceptional effectiveness across all tested conditions, achieving 80–90% reduction in surface roughness parameters regardless of initial layer height or material composition. The most dramatic improvement occurred for the highest layer height (0.52 mm), where Rz decreased from 153.42 μm to 18.03 μm—an 88.25% reduction.

**Figure 9:**
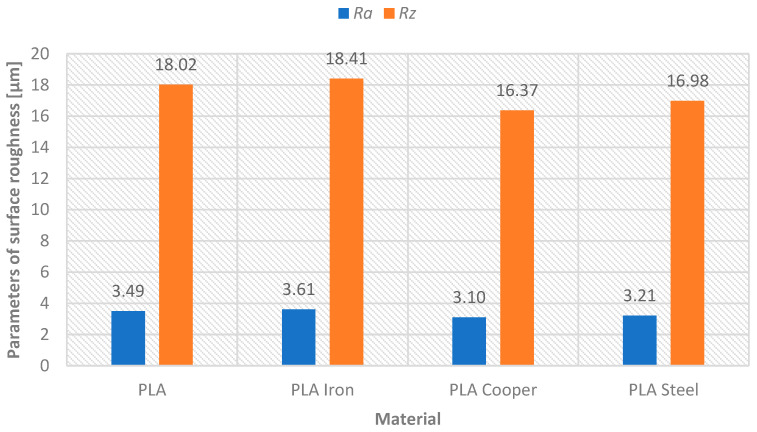
Graphical representation—applied materials versus surface roughness parameters Ra and Rz after sandblasting surface treatment.

**Figure 10:**
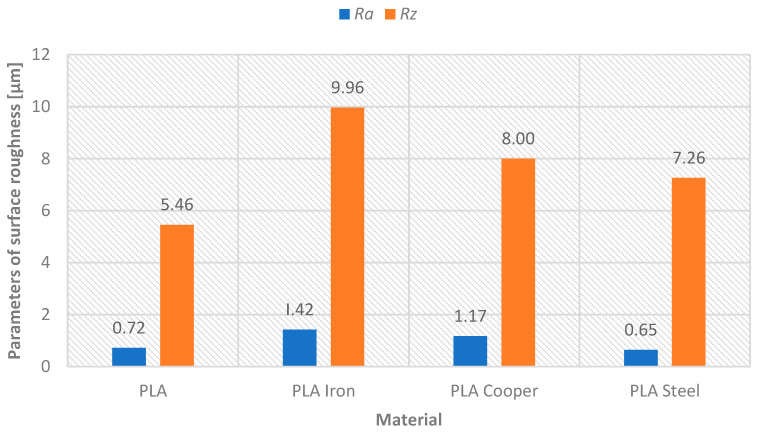
Graphical representation—applied materials versus surface roughness parameters Ra and Rz after sanding.

Model B evaluation focused on the aesthetic and functional characteristics of metal-filled PLA composites (Steel, Iron, Bronze) compared to pure PLA; all printed at 0.52 mm layer height for optimal efficiency. After sandblasting, surface roughness values showed minimal variation between materials (Rz: 16.37–18.41 μm, Ra: 3.10–3.61 μm), indicating that metal additives do not significantly impact sandblasting effectiveness—Figure 9. However, subsequent grinding revealed material dependent behavior: pure PLA and PLA Steel achieved superior grinding results with 79.37% and 79.75% improvements in Ra, respectively (final values 0.72 μm and 0.65 μm), while PLA Iron and Bronze showed reduced grinding effectiveness (60.66% and 62.26% improvements—see Table 4, final Ra values 1.42 μm and 1.17 μm)—see Figure 10.

**Figure 11:**
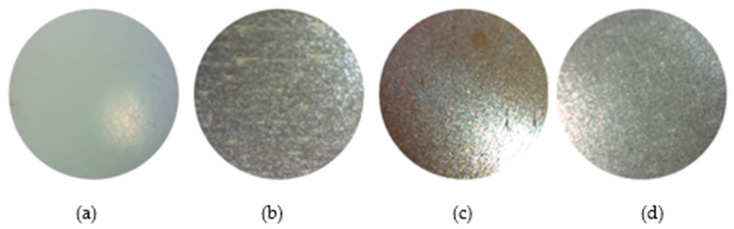
Surface details (model B) after grinding process: (**a**) PLA, (**b**) PLA Iron, (**c**) PLA Bronze, (**d**) PLA Steel at 10× magnification.

These results validate the methodology for applications requiring precision surface finishes, expanding FFF technology applicability from prototyping into functional part production for decorative and design industries where authentic metallic appearance is required without the cost and complexity of traditional metal processing—see Figure 11.

## 5. Conclusions

This research demonstrates the effectiveness of post-processing methods in achieving an authentic appearance of metal surfaces on manufactured parts, confirming a cost-effective alternative to traditional metal fabrication for design and prototyping applications. The following key findings and contributions have been established:

- Optimization of surface roughness through a strategic approach to post-processing: Post-processing methods achieved a consistent reduction of 80–90% in surface roughness parameters (Ra, Rz) regardless of the initial print layer height, demonstrating the robustness of this methodology in different production settings. The most significant improvement occurred at a layer height of 0.52 mm, where Rz decreased from 153.42 μm to 18.03 μm (a reduction of 88.25%).
- Achieving an authentic metallic appearance through material-specific reactions: Metal-containing PLA composites (steel, iron, bronze) exhibited characteristic material-dependent behavior during subsequent processing, with a final surface quality (Ra: 0.65–1.42 μm) comparable to conventional manufacturing processes, including steel drilling and light—tempering operations. PLA Steel achieved a mirror-like appearance of polished steel, PLA Bronze developed traditional patina effects, and PLA Iron created an authentic cast iron texture with controlled aging. The controlled oxidation observed in PLA Iron and Bronze is a desirable aesthetic property for design applications, creating authentic aged metal surfaces that would normally require expensive chemical treatment or years of natural aging on real metals.

Design flaws (elephant foot, interlayer adhesion failures) limit its use to decorative and low-stress components where appearance is more important than mechanical properties. The thermal sensitivity of PLA during grinding requires temperature control, while the variability of metal particle distribution affects surface consistency, especially in iron and bronze composites, where harder particles cause local surface finishing problems.

Future research directions should explore advanced composite compositions with higher metal content to increase visual authenticity and reduce the visibility of the plastic matrix. Hybrid surface treatment processes combining mechanical and chemical treatments offer the potential to achieve a premium metallic appearance consistent with product standards. The development of accelerated aging protocols could enable predictable patination timing and controlled aging patterns for historical restoration. Large-format printing capabilities would help in the integration of multiple materials, allowing for the combination of different metal finishes in individual parts; thus, this method shows significant potential for complex design applications.

## Figures and Tables

**Table 1:** Selected Properties of the Used Materials.

Material	PLA	PLA Iron	PLA Steel	PLA Bronze
Melting point	165 °C	155 °C	155 °C	155 °C
Density	1.24 g/cm^3^	1.85 g/cm^3^	2.3 g/cm^3^	2.3 g/cm^3^
Filament Diameter	1.75 mm	1.75 mm	1.75 mm	1.75 mm
Recommended Hot-End Temperature	195–205 °C	192 °C	198 °C	195 °C
Recommended Bed Temperature	60 °C	60 °C	60 °C	60 °C
Maximum Particle Size of Additives	–	250 µm	250 µm	250 µm
Type of Additives (Form)	–	Powder	Powder	Powder

**Table 2:** Input parameters for model A/model B.

	Model A	Model B
Material(s)	PLA	PLA, PLA Iron, PLA Steel, PLA Bronze
Hot-End Temperature	205 °C	205 °C
Bed Temperature	40 °C	40 °C
Print layer height	0.22/0.32/0.42/0.52 mm	0.52 mm
Print speed	25 mm/s	25 mm/s

**Table 3:** Comparison of surface roughness parameters before and after sandblasting (percentage difference indicated).

Layer height	0.22 mm		0.32 mm		0.42 mm		0.52 mm	
Parameter [µm]	Ra	Rz	Ra	Rz	Ra	Rz	Ra	Rz
Before process	16.89	76.69	23.14	102.36	29.23	129.87	34.61	153.42
After process	2.62	14.52	3.1	15.48	3.14	16.83	3.50	18.03
Difference [%]	84.50	81.06	87.01	84.87	89.24	87.04	89.89	88.25

**Table 4:** Comparison of surface roughness parameters after sandblasting/grinding—model B (indicated percentage difference).

Material	PLA		PLA Iron		PLA Bronze		PLA Steel	
Parameter [µm]	Ra	Rz	Ra	Rz	Ra	Rz	Ra	Rz
After sandblasting	3.49	18.02	3.61	18.41	3.10	16.37	3.21	16.98
After grinding	0.72	5.46	1.42	9.96	1.17	8.00	0.65	7.26
Difference [%]	79.37	69.7	60.66	45.9	62.26	51.13	79.75	57.24

## Data Availability

The raw data supporting the conclusions of this article will be made available by the authors upon request.

## Abbreviations

The following abbreviations are used in this manuscript:

ABS	Acrylonitrile-butadiene-styrene
CAD	Computer Aided Design
DM	Digital Microscopy
FDM	Fused Deposition Modelling
FFF	Fused Filament Fabrication
ME	Material Extrusion
PBF	Powder Bed Fusion
PLA	poly (lactic acid)
SEM	Scanning Electron Microscopy
SLM	Selective Laser Melting
SLS	Selective Laser Sintering
STL	Standard Tessellation Language
THF	Tetrahydrofuran
Ra	Arithmetical Mean Deviation of the Profile
Rz	Maximum Profile Height
